# Adventitial Collagen Cross-Linking by Glutaraldehyde Reinforcing Human Saphenous Vein - Implication for Coronary Artery Bypass Grafting

**DOI:** 10.21470/1678-9741-2020-0587

**Published:** 2022

**Authors:** Changcheng Liu, Duanduan Chen, Zhenfeng Li, Huanming Xu, Chengxiong Gu

**Affiliations:** 1 Department of Cardiac Surgery, Beijing Anzhen Hospital, Capital Medical University, Beijing, People’s Republic of China; 2 Beijing Institute of Heart, Lung and Blood Vessel Diseases, Beijing, People’s Republic of China; 3 Department of Biomedical Engineering, School of Life Science, Beijing Institute of Technology, Beijing, People’s Republic of China; 4 Key Laboratory of Convergence Medical Engineering System and Healthcare Technology, the Ministry of Industry and Information Technology, Beijing, People’s Republic of China

**Keywords:** Saphenous Vein, Cross-Linking, Preservation, Biological, Elastic Modulus, Staining and Labeling, Collagen, Endothelium, Coronary Artery Bypass, Nitric Oxide Synthase Type III.

## Abstract

**Introduction:**

A weak venous wall is one of the major reasons contributing to vein graft failure after coronary artery bypass grafting (CABG). We investigated whether adventitial collagen cross-linking by glutaraldehyde reinforces venous wall, preserving the endothelium of veins during high-pressure distention.

**Methods:**

Human saphenous veins (SVs) were collected from 40 patients undergoing CABG, and adventitia cross-linking was performed with 0.3% glutaraldehyde for five minutes. The cross-linked SVs were accessed by biodegradation assay, immunofluorescent staining, and tensile test. Native SVs and cross-linked SVs from another 20 patients received the 200 mmHg pressure distention for two minutes. Pressure-induced injury of SVs were accessed by immunohistochemistry and electron microscopy.

**Results:**

Time to digestion was 97±13 minutes for native SVs and 720±0 minutes for cross-linked SVs (P<0.05). After adventitial cross-linking, the collagen I fibres of the vein remarkably presented with compact and nonporous arrangement. In the high-stretch region (stretch ratio 1.4-1.8), the Young’s elastic modulus of stress-stretch ratio curve in cross-linked SVs was larger than that in native SVs (13.88 vs. 5.83, P<0.05). The cross-linked SVs had a lower extent of endothelial denudation without fibre fracture during high-pressure distension than native SVs. Comparing with the non-cross-linked SVs, the percentage of endothelial nitric oxide synthase staining length on the endothelium of cross-linked SVs was significantly preserved after high-pressure distension (85.2% vs. 64.7%, P<0.05).

**Conclusion:**

Adventitial collagen cross-linking by glutaraldehyde reinforced venous wall by increasing stiffness and decreasing extensibility of SVs and mitigated the endothelial damage under high-pressure distension.

**Table:** 

Abbreviations, acronyms & symbols			
A	= Abluminal surface	SEM	= Scanning electron microscopy
CABG	= Coronary artery bypass grafting	SMC	= Smooth muscle cells
DAPI	= 4’,6-diamidino-2-phenylindole	SVG	= Saphenous vein graft
eNOS	= Endothelial nitric oxide synthase	SVs	= Saphenous veins
GA	= Glutaraldehyde	UK	= United Kingdom
IH	= Intimal hyperplasia	USA	= United States of America
L	= Luminal surface	WSS	= Wall shear stress
PBS	= Phosphate buffered saline		

## INTRODUCTION

Coronary artery disease is the most common cause of morbidity and mortality worldwide. Although coronary artery bypass grafting (CABG) with total arterial grafts is preferred as optimal surgical therapy, using total arterial grafts is limited because of the length and number of available segments. Therefore, the autologous saphenous vein graft (SVG) remains the most widely used bypass conduit in CABG. However, 15-25% of SVGs fail one year after their implantation^[1^,^2]^. SVG failure is primarily related to intimal hyperplasia (IH), which reflects the SVG remodelling to injuries related to overdistention and endothelial denudation under the arterial environment^[3]^. Due to the weak venous wall, the SVG remodelling is induced by the low wall shear stress (WSS) and the high wall tension after exposing to pulsatile arterial pressure^[4]^.

The collagen, elastin, and smooth muscle cells (SMC) of venous wall are the major components responsible for its biomechanical properties. The adventitia of venous wall is mainly composed of collagen fibers (majorly collagen I) in a loose, wave-like aligning longitudinally^[5]^. Collagen fibers are a major structural component of extracellular matrix^[6]^. The consequence of weak venous wall results from the lack of the equilibrium between the synthesis and the degradation of the extracellular matrix^[7^,^8]^. Logically, stabilizing adventitial collagen may improve the biomechanical properties of SVG. Cross-linking, as a procedure of tissue fixation, has been used to improve the mechanical properties and in vivo stability of bioprosthetic implants^[9]^.

Glutaraldehyde (GA) is among the strongest known cross-linking agents^[10]^. It can react predominantly with the primary amine groups of lysine and hydroxylysine residues in adjacent collagen form intermediate Schiff’s bases, which then convert loose fiber monomers to stable fiber polymers^[11^,^12]^. Our previous study has indicated that adventitial collagen cross-linking by GA reduced acute overdistention and IH of vein graft in a rabbit arteriovenous graft model^[13]^. And we are also interested in the effect of adventitial collagen cross-linking by GA on the human SVG used for CABG. No data are available on biomechanical properties of SVG in relation to the cross-linking reinforced venous wall. The goal of this study is to investigate the changes of the microstructure and biomechanical properties of SVG with adventitial collagen cross-linking by GA and to evaluate the tolerance of cross-linked vein under high-pressure distension.

## METHODS

All procedures performed in this study involving human participants were in accordance with the ethical standards of the research ethics committee of Beijing Anzhen Hospital (approval number: 2017050X) and with the 1964 Helsinki declaration and its later amendments. Informed consents were obtained from all individuals.

### Preparation of the Great Saphenous Vein

The saphenous veins (SVs) from patients (age: 62±10 years; male: 80%) who underwent scheduled CABG were studied. Exclusion criteria included the following: (1) varicose veins; (2) diabetes mellitus; (3) peripheral arterial diseases; and (4) corticosteroid therapy within three months. A 5-cm long vein sample from each patient was harvested by the no-touch technique. After harvesting, the samples were stored in the heparinized saline (200 ml saline+2500 U heparin) at room temperature (22-25°C) and underwent subsequent tests.

### Adventitial Cross-Linking by GA

A total of 40 SVs segments were collected. Each SV segment was divided into two equal portions; one was cross-linked by GA, while the other served as the control. Thus, each segment from the same patient was divided into the cross-link group (n=40) and control group (n=40). Before cross-linking, the fat and excess connective tissue were removed carefully from the SV. The SV in each group was ligated by 3-0 silk sutures at both ends and was filled with saline just to maintain its tubular shape. The adventitial collagen of the SVs was then cross-linked with GA solution (0.3% w/v in 0.2 M phosphate buffered saline [PBS]) for five minutes in a 100-ml organ bath at room temperature. All cross-linked SVs were then rinsed with 0.2 M PBS for three times and one minute each time. The control SVs were only immersed in 0.2 M PBS at the same time. The intervals between SV harvesting and cross-linking was < 30 minutes. After cross-linking, all vein segments were stored in Krebs solution at 4°C and were transported to the laboratory for the histopathological and biomechanical studies. The Krebs solution was composed of NaCl 118, KCl 4.7, CaCl_2_ 2.5, KH_2_PO_4_ 1.18, MgSO_4_ 0.57, NaHCO_3_ 14.2, and glucose 5.5 (in mM).

### Cross-Linking Assessment

Biodegradation Assay: To quantitatively demonstrate collagen cross-linking in an ex vivo setting, the 2-mm segments from two groups were immersed in collagenase solution (0.1% w/v in 0.2 M PBS) (Sigma-Aldrich, St. Louis, Missouri, United States of America [USA]) and placed in an incubator at 37℃. Time to complete digestion was measured.

Immunofluorescent Staining: To visualize the ultrastructural change of venous wall after cross-linking, the immunofluorescent staining of frozen SV sections was performed. The native and adventitial cross-linked SVs were embedded in a frozen section embedding agent (optimal cutting temperature [or OCT] compound) and then cut into 7-µm thickness onto glycerinum-coated glass slides using a freezing microtome (CM1950, Leica, Germany) at -20°C. These frozen sections were incubated with primary antibodies: polyclonal anti-collagen I antibody (ab34710, Abcam, Cambridge, United Kingdom [UK]) with a dilution of 1/200 in 3% PBS for 12 hours at 4°C. The secondary antibody used was goat anti-rabbit Alexa Fluor^®^ 594 (1:1000, 2 µg/ml, Invitrogen; Carlsbad, California, USA). 4’,6-diamidino-2-phenylindole (DAPI) was used as a nuclear counterstain. The stained samples were visualized using a Leica OMI4000-B microscope and analysed by NIS-Elements AR 4.10 software (Nikon, Tokyo, Japan).

Tensile Test: The uniaxial stretch test was performed^[14]^. The SV segments from two groups were assayed within 12 hours after harvesting. The segments were cut into approximately 2 mm wide rings. The ring was cut vertically and unfolded to the venous stripe. Waterproof black ink markers were used on the surface to record local displacement. The stretch ratio was calculated by the local displacement between the centres of markings. The Cauchy stress was converted from the force signal during the stretch. During tensile testing, all venous stripes were immersed in 37°C calcium-free Krebs solution with continuously gassing 95% O_2_ and 5% CO_2_ in a 200-ml organ bath. Preconditioning stretch was performed by moving one of the clamps 2.5% of the total distance between the two clamp ends at a rate of 0.05 mm/s five times. Then, the venous stripe was stretched at 0.05 mm/s until venous failure. Considering that different regions may have different wall thicknesses, three rings ensured successful testing from different regions in each segment. The stress-stretch data were stored in a computer for offline analysis. The stress-stretch relationship was analysed by Cauchy stress-stretch ratio curves that reflect biomechanical behaviour. Additionally, Young’s elastic modulus was calculated to determine the material’s stiffness.

Data processing was performed by the following equations:

$$\sigma =\frac{F}{WT}=\frac{F}{W_{0}T_{0}}\lambda$$

in which σ and λ stand for the Cauchy stress and stretch ratio, respectively; *F* is the stretching force; and *W*_0_ and *T*_0_ stand for the width and thickness at rest, respectively^[15]^.

A modified Mooney-Rivlin strain energy density function^[14]^ was used to characterize the stretch-stress relationship of SVs.

$$W=C_{1}\left({\tilde{I}}_{1}-3\right)+D_{1}\left[e^{D_{2}\left({\tilde{1}}_{1}-3\right)}-1\right]+\kappa (J-1)$$

in which *I*_1_ and *J* stand for the first invariant and Jacobian of the deformation gradient tensor, respectively; *D*_1_ and *D*_2_ are material constants, and ? is the Lagrange multiplier for the incompressibility.

Moreover, the Young’s elastic modulus was defined as

$$E(\lambda )=\frac{d\sigma }{d\lambda }$$

### High-Pressure Distension and Histological Analysis

In order to test whether adventitial cross-linked SV was better tolerated with arterial pressure, a high-pressure distension test was carried out. The SV segments (approximately 10 cm) from additional 20 patients were harvested by no-touch technique and immediately stored in Krebs solution. Each segment was divided into three equal portions and randomly assigned to one of three groups: non-distended (negative control), adventitial GA cross-linking for five minutes with 200 mmHg of pressure distension for two minutes (study group), or no cross-linking with 200 mmHg of pressure distension for two minutes (positive control). The proximal end of the SV was ligated with 3-0 silk sutures, and the distal end was cannulated with an olive-tipped needle. A 50-mL handheld syringe with pressure gauge (0-300 mmHg) was connected to the olive-tipped needle. Distension was performed by pumping saline with syringe. The interval between harvest and cross-linking with high-pressure distension was maintained within 30 minutes. Structural damage related to high-pressure distension was evaluated by immunohistochemistry and scanning electron microscopy (SEM).

For immunohistochemistry, the samples were fixed in 10% neutral formalin before dehydrating in a graded series of alcohol, clearing in xylene, and embedding in paraffin. Five-micron sections perpendicular to the long axis of the SV were cut from paraffin-embedded tissue blocks onto slides and were dewaxed and rehydrated by passing through graded alcohol and rinsing in water. Tissue antigen was retrieved using citrate buffer (pH 6) at 95°C for 15 minutes. Endogenous peroxidase was blocked by immersing slides in 3% hydrogen peroxide for 15 minutes. These sections were incubated with primary antibodies against endothelial nitric oxide synthase (eNOS) (ab5589, Abcam, Cambridge, UK) for one hour at room temperature. Goat anti-mouse immunoglobulin G (ab6789, Abcam, Cambridge, UK) was used as a secondary antibody at 10 ng/mL. Negative controls were achieved by omitting incubation with the primary antibody. Three discrete slides of each sample were observed using an Eclipse Ni-E light microscope (Nikon, Tokyo, Japan).

For SEM, the samples were fixed in 2.5% GA for one hour at 4°C. After fixation, samples were rinsed in 0.1 M PBS (pH 7.2) and fixed in 1% osmium tetroxide solution for one hour at 4°C. After rinsing in 0.1 M PBS, the samples underwent progressive dehydration in 50%, 70%, and 90% ethanol solutions at 4°C and 100% ethanol solutions three times at room temperature. The samples were then immersed in pure tert-butyl alcohol overnight and freeze-dried, sputter coated with gold/palladium, and visualized using FEI Inspect S50 electron microscope (FEI, USA).

### Statistical Analysis

Continuous variables and categorical data are expressed as the mean ± standard deviation and percentage, respectively. Young’s elastic modulus was used for statistical evaluation of the stress-stretch relationship. Student’s t-test or analysis of variance test was used to address non-paired samples for the comparison of normally distributed parameters, and Wilcoxon rank sum test was used for the comparison of non-parametric variables. Data were analysed using SAS version 9.4 (SAS Institute, Cary, North Carolina, USA). A difference with P<0.05 was considered significant.

## RESULTS

### Cross-Linking Assessment of SVs

**Fig. 1:**
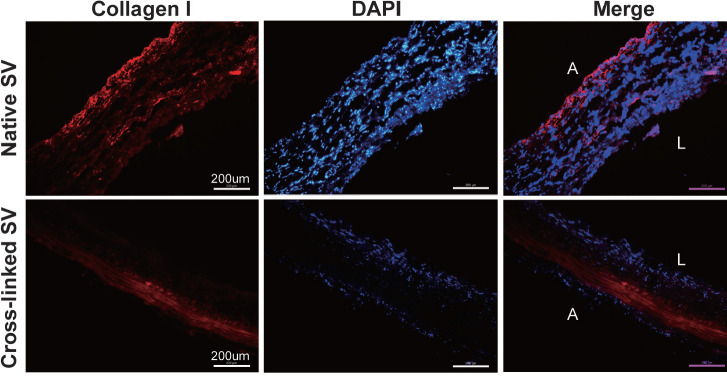
Collagen I immunofluorescent staining of frozen section. The degree of cross-linking was identified by comparing with different immunofluorescent images (magnification, ×200). A=abluminal surface; DAPI=4’,6-diamidino-2-phenylindole; L=luminal surface; SV=saphenous vein

The ultrastructural change of SVs after adventitial cross-linking was accessed by immunofluorescence of frozen section (Figure 1). In the control group, collagen I fibres were typically arranged in an unconsolidated and porous manner. However, collagen I fibres were remarkably presented with compact and nonporous arrangement in the cross-link group. In addition, compact collagen I fibres were majorly located in the adventitia according to the merge micrographs. The layers adjacent to the luminal surface seemed to have similar morphology in both crosslinked groups and the control group according to the DAPI micrographs.

The digestion time was 97±13 minutes for native SVs (n=40) and 720±0 minutes for adventitial cross-linked SVs (n=40). The experiments were terminated after 12 hours, at which time none of the adventitial cross-linked samples were fully digested in contrast to the native samples, which were all fully digested (*P*<0.05).

### Biomechanical Property Changes of Cross-Linked SVs

**Fig. 2:**
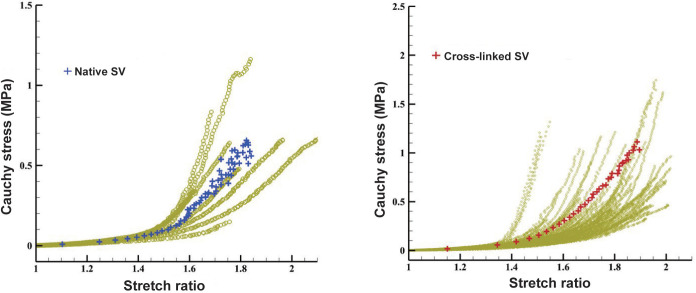
Stain-stretch relationships of the native and cross-linked saphenous veins (SVs). In the high-stretch region (stretch ratio: 1.4-1.8), the circumferential stress in the cross-linked SV was higher than that in the native SV indicating decreased extensibility and reinforced venous wall of the SVs.

The tensile test showed the nonlinear Cauchy stress-stretch ratio relationships of the SV in the control and cross-linked SVs (Figure 2). In the low-stretch region (stretch ratio: 1.0-1.4), the circumferential stress in the cross-linked SV was similar to that in the native SV. However, in the high-stretch region (stretch ratio: 1.4-1.8), the circumferential stress in the cross-linked SV was higher than that in the native SV. The constant parameters from the tensile analysis of Cauchy stress-stretch ratio curves are shown in Table 1. In the high-stretch region, the Young’s elastic modulus of stress-stretch ratio curve in cross-linked SVs was larger than that in native SVs (13.88 *vs*. 5.83, *P*<0.05).

**Table 1:** The mechanical constants of saphenous veins (SVs) with or without cross-linking.

Variables	Low-stretch region (σ=a ɛ+b)			High-stretch region (σ=c ɛ+d)		
	Parameter	Native SV	Cross-linked SV	Parameter	Native SV	Cross-linked SV
Cross-linking for 5 minutes	A	0.3089	0.5450	c	5.8292	13.8800
	B	-0.3434	-0.6240	d	-7.6862	-18.7300
	R-square	0.8672	0.9217	R-square	0.5505	0.9143

σ=Cauchy stress; ɛ=stretch ratio; a and c=Young’s elastic modulus; R-square=coefficient of determination

### Ultrastructural Changes of Cross-Linked SV Under High-Pressure Distension

**Fig. 3:**
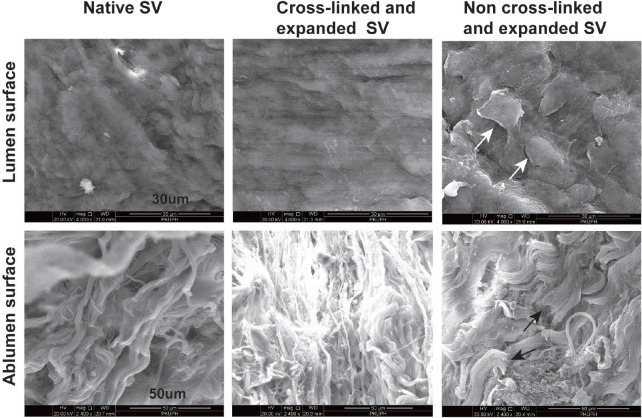
Scanning electron microscopy of human saphenous vein (SV). The ultrastructure of luminal and abluminal surfaces was observed in SVs with different processing (luminal surface magnification, ×4000; abluminal surface magnification, ×2400). The white arrows show patches of endothelium containing denuded and curly cells. The black arrows show fibre fracture.

The SEM images in Figure 3 show the luminal and abluminal surfaces of SV with different processing. In the native SVs, the luminal surface typically revealed an intact, smooth endothelium with mildly overlapping boundaries of the single endothelial cells; and the abluminal surface was typically wavy with incompact distribution of collagenous fibres. In the SVs that underwent adventitial cross-linking for five minutes with distension under 200 mmHg of pressure, the luminal surface largely reserved an intact, smooth endothelium with little overlapping boundaries of the single endothelial cells; the abluminal surface revealed higher fibre junctions with compact distribution than native segments. However, in the SVs that suffered 200 mmHg of pressure distension without adventitial cross-linking, luminal surface integrity was remarkably damaged, and patches of endothelium containing denuded and curly cells with distinct intercellular space were presented; the structure of fibres in the abluminal surface was destroyed, presenting fibre fracture due to high-pressure distension.

**Fig. 4:**
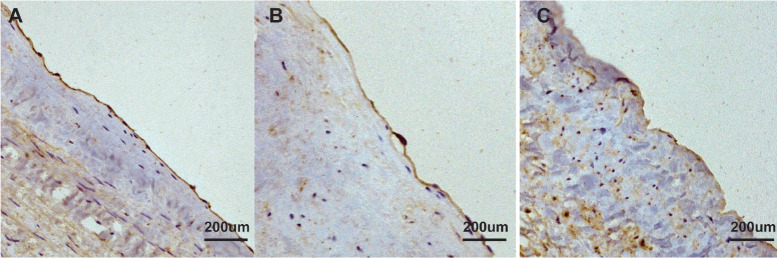
Immunohistochemistry staining of human saphenous vein (SV). The endothelial nitric oxide synthases (brown) were stained along with the luminal surface. Adventitial cross-linking preserved the endothelial integrity largely by comparing immunohistochemistry images (magnification, ×200) in different groups. A) Native SV; B) cross-linked and expanded SV; C) non-cross-linked and expanded SV.

**Fig. 5:**
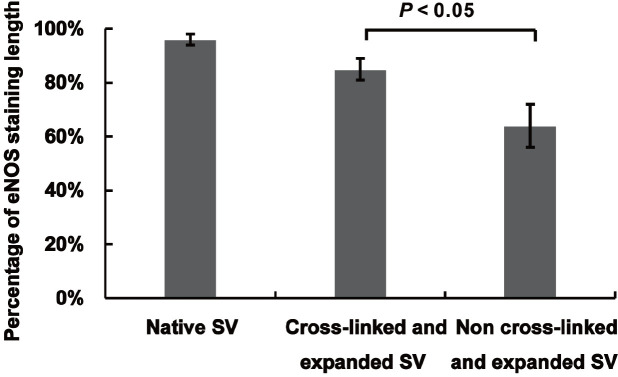
The quantitative comparison of endothelial nitric oxide synthase (eNOS) staining of human saphenous vein (SV). Adventitial cross-linking significantly preserved eNOS staining after high-pressure distension which indicated adventitial cross-linking protected the endothelial integrity under high-pressure distension.

The endothelial integrity was assessed by immunohistochemical staining for eNOS (Figure 4). Strong and continuous staining for eNOS was shown along the endothelial surface in the native SVs. In the SVs receiving adventitial cross-linking for five minutes with distension under 200 mmHg of pressure, the endothelial surface exhibited almost intact staining. However, the SVs without adventitial cross-linking that suffered 200 mmHg of pressure distension remarkably revealed patchy staining with light dyeing. Figure 5 shows that comparing with the non-cross-linked SVs, the percentage of eNOS staining length on the endothelium of cross-linked SVs was significantly preserved after high-pressure distension (85.2% *vs*. 64.7%, *P*<0.05).

## DISCUSSION

The change of biological environment from venous system to arterial system is one of the major triggers leading to SVG pathological remodelling and restenosis. Due to the vein being a volume-related organ, the weak venous wall failed to adapt itself to high distending pressures following implantation into the arterial circulation. Additionally, pressure-induced vein distension could cause structural injury, mainly indicating endothelial denudation^[16^,^17]^. The integrity disruption of endothelial surface and exposure of thrombogenic subluminal layer may contribute to IH and SVG failure by activating inflammatory response^[18]^ and disturbing endothelial cell function^[19]^ and SMC^[20]^. Thus, reinforcing the venous wall may control the overdistension of the SVG in the arterial circulation and then mitigate pathological remodelling of the SVG. In this pilot study, we tried GA cross-linking to reinforce the venous wall.

Previous studies have demonstrated that chemical cross-linking improved the biomechanical properties and in vivo stability of implants^[6^,^9]^. Consistent with these studies, our study also indicated that adventitial cross-linking decreased the extensibility of the SV according to the stress-stretch curves. According to the law of Laplace (σ_ϴ_=P*r/t, σ_ϴ_ is circumferential stress, *P* is transmural pressure, *r* is inner radius, and *t* is wall thickness), cross-linking prevented increasing *r* and decreasing *t* by reducing the extensibility of the SV. Moreover, σ_ϴ_ was smaller in cross-linked SVG than that in native SVG following CABG. Hence, decreasing the extensibility of the SV could improve the ability to resist pressure-induced geometric distension. However, geometric distension contributed to cause metabolic injury of SMC^[21]^, subsequently increasing SMC proliferation^[22]^. To some extent, cross-linking may mitigate the pathological remodelling of SVG under arterial circulation.

Furthermore, adventitial cross-linking also increased the stiffness of the SV, thereby decreasing its compliance. The decreased compliance could avoid enlarging inner radius (*r*) and maintain relatively constant local blood flow rate (*Q*) under arterial circulation. According to *WSS=4Q/r^^3^^*, cross-linking maybe prevent lower WSS at the cross-linked SVG than that at the native SVG following CABG. Previous studies have indicated that low WSS could promote IH of SVG^[23^-^25]^. Thus, when being grafted to a coronary artery, native SVG maybe suffer more serious pathological remodelling than cross-linked SVG. And Salinas et al.^[26]^ has demonstrated that the photochemical cross-linking in adventitial collagen could limit acute dilatation and suppress IH of vein grafts in an animal model.

The degree of cross-linking was controlled by either increasing GA concentration or lengthening the cross-linked time. The typical concentration of GA was 0.6% w/v for tissue cross-linking^[6]^, and cross-linked time was counted by hours or days^[12^,^27]^ in the preparation process of bioprosthetic implants. In our study, we selected the lower GA concentration (0.3% w/v) with shorter cross-linked time (five minutes). A previous study confirmed that GA primarily cross-linked the surface of the collagen fibres and created a polymeric network, which hindered the further cross-linking of the interstitium of the fibre^[28]^. We performed cross-linking with a lower GA concentration and shorter time only in the abluminal surface to keep the bioactivity of the endothelium. Our immunofluorescence results also indicated that tissue cross-linking mainly occurred at the adventitia of the SV, and the density of collagen was significantly increased in the cross-linked SV compared to that in the native SV, which implicated that adventitial cross-linking perhaps do not affect endothelial function.

The endothelium plays an essential role in preserving physiological function of vessels. Hence, keeping the structural integrity of the endothelium is very important in the SVG. Viaro et al.^[16]^ confirmed that exposure to 300 mmHg for 15 seconds could cause nearly complete endothelial denudation in human SV. Our results indicated that adventitial cross-linking reduced the degree of endothelial denudation of the SV during 200 mmHg of pressure distension for two minutes. Typically, SVG underwent the drastic pressure change from venous circulation (approximately 10 mmHg) to arterial circulation (approximately 100 mmHg). Necla et al.^[29]^ indicated that distending pressure > 75 mmHg might contribute to SVG failure. The adventitial cross-linking might play a protective role in moderating SVG failure by reinforcing the venous wall and preventing endothelial denudation.

### Limitations

Some limitations should be recognized in this study. Firstly, as an ex vivo study, although adventitial cross-linking implied a change of biomechanical properties and protective effect in the endothelium, a pulsatile perfusion model with SVG is still required to test these effects of adventitial cross-linking. Secondly, adventitial collagen cross-linking by GA damaged the perivenous vasa vasorum. However, no data on the exact effect of vasa vasorum on IH and graft patency after implantation was found^[30]^. Thirdly, this study is a pilot and qualitative research. Systematic study should be conducted to explore the optimal degree of adventitial cross-linking.

## CONCLUSION

Adventitial collagen cross-linking by GA reinforced venous wall by increasing the stiffness and decreasing the extensibility of SVG and mitigated the endothelial damage under high-pressure distension. Adventitial cross-linking may be a simple and cost-effective approach to reinforce the venous wall, and it appears to protect SVG from the arterial circulation.

**Table:** 

Authors’ Roles & Responsibilities	
CL	Substantial contributions to the acquisition and analysis of data for the work; drafting the work; final approval of the version to be published
DC	Substantial contributions to the acquisition and analysis of data for the work; final approval of the version to be published
ZL	Substantial contributions to the acquisition and analysis of data for the work; final approval of the version to be published
HX	Substantial contributions to the acquisition and analysis of data for the work; final approval of the version to be published
CG	Substantial contributions to the design of the work; revising the work; final approval of the version to be published

## References

[1] Windecker S, Kolh P, Alfonso F, Collet JP, Cremer J, Authors/Task Force members (2014). 2014 ESC/EACTS guidelines on myocardial revascularization: the
task force on myocardial revascularization of the European society of
cardiology (ESC) and the European association for cardio-thoracic surgery
(EACTS) developed with the special contribution of the European association
of percutaneous cardiovascular interventions (EAPCI). Eur Heart J.

[2] Harskamp RE, Lopes RD, Baisden CE, de Winter RJ, Alexander JH. (2013). Saphenous vein graft failure after coronary artery bypass
surgery: pathophysiology, management, and future directions. Ann Surg.

[3] Davies MG, Hagen PO. (2011). Reprinted article "Pathophysiology of vein graft failure: a
review". Eur J Vasc Endovasc Surg.

[4] Morrow D, Sweeney C, Birney YA, Guha S, Collins N, Cummins PM (2007). Biomechanical regulation of hedgehog signaling in vascular smooth
muscle cells in vitro and in vivo. Am J Physiol Cell Physiol.

[5] Canham PB, Finlay HM, Boughner DR. (1997). Contrasting structure of the saphenous vein and internal mammary
artery used as coronary bypass vessels. Cardiovasc Res.

[6] Chandran PL, Paik DC, Holmes JW. (2012). Structural mechanism for alteration of collagen gel mechanics by
glutaraldehyde crosslinking. Connect Tissue Res.

[7] Sansilvestri-Morel P, Rupin A, Badier-Commander C, Kern P, Fabiani JN, Verbeuren TJ (2001). Imbalance in the synthesis of collagen type I and collagen type
III in smooth muscle cells derived from human varicose veins. J Vasc Res.

[8] Badier-Commander C, Verbeuren T, Lebard C, Michel JB, Jacob MP. (2000). Increased TIMP/MMP ratio in varicose veins: a possible
explanation for extracellular matrix accumulation. J Pathol.

[9] Goissis G, Yoshioka SA, Braile DM, Ramirez VD. (1998). The chemical protecting group concept applied in crosslinking of
natural tissues with glutaraldehyde acetals. Artif Organs.

[10] Richards FM, Knowles JR. (1968). Glutaraldehyde as a protein cross-linkage reagent. J Mol Biol.

[11] Billiar KL, Sacks MS. (2000). Biaxial mechanical properties of the natural and glutaraldehyde
treated aortic valve cusp--Part I: Experimental results. J Biomech Eng.

[12] Wine Y, Cohen-Hadar N, Freeman A, Frolow F. (2007). Elucidation of the mechanism and end products of glutaraldehyde
crosslinking reaction by X-ray structure analysis. Biotechnol Bioeng.

[13] Liu C, Yu W, Chen D, Shi Y, Li Z, Gu C. (2020). Adventitial collagen crosslink reduces intimal hyperplasia in a
rabbit arteriovenous graft model. J Surg Res.

[14] Teng Z, Zhang Y, Huang Y, Feng J, Yuan J, Lu Q (2014). Material properties of components in human carotid
atherosclerotic plaques: a uniaxial extension study. Acta Biomater.

[15] Teng Z, Feng J, Zhang Y, Sutcliffe MP, Huang Y, Brown AJ (2015). A uni-extension study on the ultimate material strength and
extreme extensibility of atherosclerotic tissue in human carotid
plaques. J Biomech.

[16] Viaro F, Capellini VK, Celotto AC, Carlotti CG, Rodrigues AJ, Reis GS (2010). Immunohistochemical evaluation of three nitric oxide synthase
isoforms in human saphenous vein exposed to different degrees of distension
pressures. Cardiovasc Pathol.

[17] Thatte HS, Khuri SF. (2001). The coronary artery bypass conduit: I. Intraoperative endothelial
injury and its implication on graft patency. Ann Thorac Surg.

[18] Khaleel MS, Dorheim TA, Duryee MJ, Durbin HE, Bussey WD, Garvin RP (2012). High-pressure distention of the saphenous vein during preparation
results in increased markers of inflammation: a potential mechanism for
graft failure. Ann Thorac Surg.

[19] Dumanski A, Sopel M, Pelczar M, Szłapka M, Kustrzycki W, Zabel M. (2007). Influence of pressure on the endothelium of the saphenous vein
coronary artery bypass graft. In Vivo.

[20] Hocking KM, Brophy C, Rizvi SZ, Komalavilas P, Eagle S, Leacche M (2011). Detrimental effects of mechanical stretch on smooth muscle
function in saphenous veins. J Vasc Surg.

[21] Angelini GD, Breckenridge IM, Butchart EG, Armistead SH, Middleton KM, Henderson AH (1985). Metabolic damage to human saphenous vein during preparation for
coronary artery bypass grafting. Cardiovasc Res.

[22] Angelini GD, Soyombo AA, Newby AC. (1991). Winner of the ESVS prize 1990. Smooth muscle cell proliferation
in response to injury in an organ culture of human saphenous
vein. Eur J Vasc Surg.

[23] Tardy Y, Resnick N, Nagel T, Gimbrone MA (1997). Jr, Dewey CF Jr. Shear stress gradients remodel endothelial
monolayers in vitro via a cell proliferation-migration-loss
cycle. Arterioscler Thromb Vasc Biol.

[24] DePaola N, Gimbrone MA, Davies PF, Dewey CF (1992). Vascular endothelium responds to fluid shear stress
gradients. Arterioscler Thromb.

[25] Poranen AK, Aubry J, Kujari H, Ekblad U. (1998). Expression of nitric oxide synthase in normal and preeclamptic
placental tissue and effects of glyceryl trinitrate and shear stress on
placental blood flow. Acta Obstet Gynecol Scand.

[26] Salinas HM, Khan SI, McCormack MC, Fernandes JR, Gfrerer L, Watkins MT (2017). Prevention of vein graft intimal hyperplasia with photochemical
tissue passivation. J Vasc Surg.

[27] Kim KM, Herrera GA, Battarbee HD. (1999). Role of glutaraldehyde in calcification of porcine aortic valve
fibroblasts. Am J Pathol.

[28] Cheung DT, Perelman N, Ko EC, Nimni ME. (1985). Mechanism of crosslinking of proteins by glutaraldehyde III.
Reaction with collagen in tissues. Connect Tissue Res.

[29] Ozturk N, Sucu N, Comelekoglu U, Yilmaz BC, Aytacoglu BN, Vezir O. (2013). Pressure applied during surgery alters the biomechanical
properties of human saphenous vein graft. Heart Vessels.

[30] Gooch KJ, Firstenberg MS, Shrefler BS, Scandling BW. (2018). Biomechanics and mechanobiology of saphenous vein
grafts. J Biomech Eng.

